# From People to *Panthera*: Natural SARS-CoV-2 Infection in Tigers and Lions at the Bronx Zoo

**DOI:** 10.1128/mBio.02220-20

**Published:** 2020-10-13

**Authors:** Denise McAloose, Melissa Laverack, Leyi Wang, Mary Lea Killian, Leonardo C. Caserta, Fangfeng Yuan, Patrick K. Mitchell, Krista Queen, Matthew R. Mauldin, Brittany D. Cronk, Susan L. Bartlett, John M. Sykes, Stephanie Zec, Tracy Stokol, Karen Ingerman, Martha A. Delaney, Richard Fredrickson, Marina Ivančić, Melinda Jenkins-Moore, Katie Mozingo, Kerrie Franzen, Nichole Hines Bergeson, Laura Goodman, Haibin Wang, Ying Fang, Colleen Olmstead, Colleen McCann, Patrick Thomas, Erin Goodrich, François Elvinger, David C. Smith, Suxiang Tong, Sally Slavinski, Paul P. Calle, Karen Terio, Mia Kim Torchetti, Diego G. Diel

**Affiliations:** a Wildlife Conservation Society, Bronx Zoo, Bronx, New York, USA; b Department of Population Medicine and Diagnostic Sciences, Animal Health Diagnostic Center, College of Veterinary Medicine, Cornell University, Ithaca, New York, USA; c Veterinary Diagnostic Laboratory, College of Veterinary Medicine, University of Illinois, Urbana, Illinois, USA; d National Veterinary Services Laboratories, Animal and Plant Health Inspection Service (APHIS), U.S. Department of Agriculture (USDA), Ames, Iowa, USA; e Department of Pathobiology, College of Veterinary Medicine, University of Illinois, Urbana, Illinois, USA; f Centers for Disease Control and Prevention, Atlanta, Georgia, USA; g Zoological Pathology Program, College of Veterinary Medicine, University of Illinois, Brookfield, Illinois, USA; h Chicago Zoological Society, Chicago, Illinois, USA; i New York State Department of Agriculture and Markets, Albany, New York, USA; j New York City Department of Health and Mental Hygiene, Queens, New York, USA; Virginia Polytechnic Institute and State University

**Keywords:** One Health, *Panthera leo*, *Panthera tigris*, SARS-CoV-2, *in situ* hybridization, lion, rRT-PCR, tiger, virus isolation, whole-genome sequencing, zoo, zoonotic infection

## Abstract

Despite numerous barriers to transmission, zoonoses are the major cause of emerging infectious diseases in humans. Among these, severe acute respiratory syndrome (SARS), Middle East respiratory syndrome (MERS), and ebolaviruses have killed thousands; the human immunodeficiency virus (HIV) has killed millions. Zoonoses and human-to-animal cross-species transmission are driven by human actions and have important management, conservation, and public health implications. The current SARS-CoV-2 pandemic, which presumably originated from an animal reservoir, has killed more than half a million people around the world and cases continue to rise. In March 2020, New York City was a global epicenter for SARS-CoV-2 infections. During this time, four tigers and three lions at the Bronx Zoo, NY, developed mild, abnormal respiratory signs. We detected SARS-CoV-2 RNA in respiratory secretions and/or feces from all seven animals, live virus in three, and colocalized viral RNA with cellular damage in one. We produced nine whole SARS-CoV-2 genomes from the animals and keepers and identified different SARS-CoV-2 genotypes in the tigers and lions. Epidemiologic and genomic data indicated human-to-tiger transmission. These were the first confirmed cases of natural SARS-CoV-2 animal infections in the United States and the first in nondomestic species in the world. We highlight disease transmission at a nontraditional interface and provide information that contributes to understanding SARS-CoV-2 transmission across species.

## Abstract (precis)

The human-animal-environment interface of severe acute respiratory syndrome coronavirus 2 (SARS-CoV-2) is an important aspect of the coronavirus disease 2019 (COVID-19) pandemic that requires robust One Health-based investigations. Despite this, few reports describe natural infections in animals or directly link them to human infections using genomic data. In the present study, we describe the first cases of natural SARS-CoV-2 infection in tigers and lions in the United States and provide epidemiological and genetic evidence for human-to-animal transmission of the virus. Our data show that tigers and lions were infected with different genotypes of SARS-CoV-2, indicating two independent transmission events to the animals. Importantly, infected animals shed infectious virus in respiratory secretions and feces. A better understanding of the susceptibility of animal species to SARS-CoV-2 may help to elucidate transmission mechanisms and identify potential reservoirs and sources of infection that are important in both animal and human health.

## INTRODUCTION

Coronaviruses are a recognized cause of disease in humans and animals (1). Among them are the viruses that cause colds in humans, multisystemic disease in domestic cats, and gastrointestinal and respiratory diseases in pigs and poultry. Coronavirus disease 2019 (COVID-19) caused by severe acute respiratory syndrome-related coronavirus (SARS-CoV-2) (2) was first reported in Wuhan, Hubei province, China at the end of December 2019 (3). Within weeks the virus spread globally, and by July 2020, over 10 million people were infected and more than 500,000 had died (https://www.who.int/emergencies/diseases/novel-coronavirus-2019; accessed 1 July 2020). Genome sequence analysis has shown SARS-CoV-2 to be most closely related to a bat coronavirus (RaTG13-2013), and horseshoe bats are currently considered the likely source of the ancestral virus from which the currently circulating SARS-CoV-2 virus was derived (4, 5). Subsequent genetic adaptation of the currently circulating virus in an intermediate animal host(s) or after human transmission has been proposed (5, 6). The exact details of how long the virus had been circulating in animals prior to transmission to people is unknown. However, a recent study suggests the virus may have been circulating in bats for several decades (7), and an early cluster of human COVID-19 cases had an epidemiological link to the Huanan Seafood Wholesale market in Wuhan where a variety of live wild animals were sold (4). The current SARS-CoV-2 pandemic and outbreaks of severe acute respiratory syndrome (SARS) and Middle East respiratory syndrome (MERS) before it raise awareness and concerns about zoonotic (animal-to-human) diseases and cross-species transmission of coronaviruses (8–11).

Given the suspected zoonotic origin of SARS-CoV-2, identifying susceptible animal species, reservoirs, and transmission routes between species is a topic of global scientific and public interest. Natural SARS-CoV-2 infections in animals have been reported in dogs, cats, and farmed mink in Hong Kong, Europe, China, and the United States (12–14). Infection in most of these cases has been linked to households or settings in which human owners or caretakers have tested positive for SARS-CoV-2 and infection from humans to animals has been presumed. Experimental inoculation studies have shown that SARS-CoV-2 infects and replicates with high efficiency in domestic cats, ferrets, and fruit bats and poorly in dogs; pigs, chickens, and ducks do not seem to support productive SARS-CoV-2 infection (15, 16). Importantly, virus shedding and horizontal transmission have been shown in cats and ferrets (15–17) following experimental inoculation.

In this study, we report natural infection of tigers (Panthera tigris) and lions (Panthera leo) with SARS-CoV-2 at the Wildlife Conservation Society’s (WCS’s) Bronx Zoo, New York, NY, and provide a detailed genomic characterization of viruses obtained from infected animals and keepers who had close contact with the SARS-CoV-2-positive animals. These were the first confirmed animal infections in the United States and occurred in March 2020, when, due to widespread community transmission (18), New York was a global SARS-CoV-2 epicenter.

## RESULTS

### Clinical investigation in affected animals.

On 27 March 2020, a 4-year-old, female Malayan tiger (*Panthera tigris jacksoni*) (Tiger 1) developed an intermittent cough and audible wheezing despite remaining eupneic. By 3 April, an additional Malayan tiger (Tiger 2) and two Amur tigers (*Panthera tigris altaica*) (Tigers 3 and 4) housed in the same building as Tiger 1 but in different enclosures and three African lions (*Panthera leo krugeri*) (Lions 1, 2, and 3) housed in a separate building developed similar respiratory signs. All animals otherwise exhibited normal behavior and activity. Clinical respiratory signs resolved in less than 5 days (3 to 5 April 2020) in all animals except Tiger 1, whose clinical signs lasted 16 days (resolution of clinical signs on 12 April 2020). An additional Amur tiger (Tiger 5) in the same building as Tigers 1 to 4 did not develop clinical respiratory disease.

### Detection of SARS-CoV-2 in affected animals.

**FIG 1:**
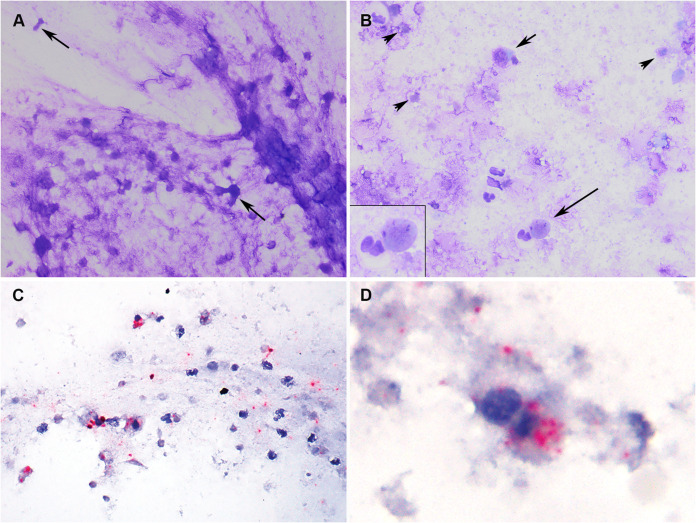
Tracheal wash cytology (A and B) and *in situ* hybridization (ISH) (C and D). Tiger 1. (A) Flocculent material from the trachea consists of stringy mucus with enmeshed degenerate cells characterized by condensed nuclei and loss of distinct cellular features (arrows). (B) Few intact cells (short arrow) and degenerate epithelial cells (long arrow) are admixed with abundant round to amorphous cellular debris and granular degenerate mucus (arrowheads). Inset: degenerate epithelial cell (upper right) with nuclear fragmentation (karyolysis) and an adjacent intact neutrophil (lower left). Modified Wright’s stain. (C and D) Incubation with SARS-CoV-2-specific probe is positive (red puncta) throughout the mucinous material, in the cytoplasm of intact and degenerate epithelial and inflammatory cells, and in cellular debris. Red chromogenic assay; hematoxylin counterstain. (Note: a software or equipment malfunction produced a faint horizontal line that may be visible in panels A and B.)

A broad diagnostic investigation was performed in Tiger 1 on 2 April, 6 days after the onset of clinical signs. This included physical examination, thoracic and abdominal radiography and ultrasonography, and collection of respiratory (nasal swab, oropharyngeal swab, and tracheal wash) and blood samples. Thoracic radiography and ultrasonography revealed small, multifocal regions of peribronchial consolidation. Cytologic examination of tracheal wash fluid identified necrotic epithelial and inflammatory cells consistent with tracheitis (Fig. 1A and B). *In situ* hybridization (ISH) colocalized SARS-CoV-2 RNA within necrotic epithelial and inflammatory cells in the fluid (Fig. 1C and D and see also Fig. S1). All respiratory samples (nasal, oropharyngeal, and tracheal wash) were negative on virus isolation for feline herpesvirus and feline calicivirus and on targeted PCR testing and metagenomic analysis for common feline pathogens (Table S1, BioProject accession no. PRJNA627354); all were positive for SARS-CoV-2 by real-time reverse transcription-PCR (rRT-PCR) using primers and probes targeting portions of the nucleocapsid (N1, N2, and N3) and envelope (E) genes (Table S2). MinION and Sanger-based sequencing of the full SARS-CoV-2 spike (S) gene, an internal region of the N gene, and the RNA-dependent RNA polymerase (RdRp) confirmed the virus in the respiratory samples (Table S3 and Data Set 1 [see “Data availability” in Materials and Methods for definitions and locations of all data sets]).

10.1128/mBio.02220-20.1 FIG S1 Negative controls for *in situ* hybridization. No staining is seen in any of the control samples, confirming specificity of the probes for SARS-CoV-2 and lack of cross-reaction with normal tiger cells and feline alphacoronaviruses. (A) Direct smears of tracheal wash sample from Tiger 1. (B) Infected Vero cells. (C) Feline enteric coronavirus-infected cells. (D) Histologic section of mesentery from a domestic cat with confirmed feline infectious peritonitis due to feline coronavirus infection. (E and F) Histologic sections of normal Malayan tiger trachea (E) and lung (F) collected prior to the emergence of SARS-CoV-2. Download FIG S1, JPG file, 1.0 MB. Copyright © 2020 McAloose et al. 2020 McAloose et al. This content is distributed under the terms of the Creative Commons Attribution 4.0 International license.

10.1128/mBio.02220-20.4 TABLE S1 Targeted feline respiratory pathogen testing in Tiger 1. Download Table S1, DOCX file, 0.01 MB. Copyright © 2020 McAloose et al. 2020 McAloose et al. This content is distributed under the terms of the Creative Commons Attribution 4.0 International license.

10.1128/mBio.02220-20.5 TABLE S2 Average rRT-PCR *C_T_* values for SARS-CoV-2 targets in respiratory samples. Download Table S2, DOCX file, 0.01 MB. Copyright © 2020 McAloose et al. 2020 McAloose et al. This content is distributed under the terms of the Creative Commons Attribution 4.0 International license.

10.1128/mBio.02220-20.6 TABLE S3 Targeted MinION amplicon sequencing in tracheal wash fluid and fecal samples. Download Table S3, DOCX file, 0.01 MB. Copyright © 2020 McAloose et al. 2020 McAloose et al. This content is distributed under the terms of the Creative Commons Attribution 4.0 International license.

Fecal samples collected opportunistically from each animal (symptomatic Tigers 1 to 4, asymptomatic Tiger 5, and symptomatic Lions 1 to 3) tested positive for SARS-CoV-2 by rRT-PCR (Table S4). Results were confirmed by amplicon sequencing (Table S3 and Data Set 2).

10.1128/mBio.02220-20.7 TABLE S4 Average rRT-PCR *C_T_* values for SARS-CoV-2 targets in fecal samples. Download Table S4, DOCX file, 0.01 MB. Copyright © 2020 McAloose et al. 2020 McAloose et al. This content is distributed under the terms of the Creative Commons Attribution 4.0 International license.

### Infectious SARS-CoV-2 detected in respiratory and fecal samples from affected animals.

**FIG 2:**
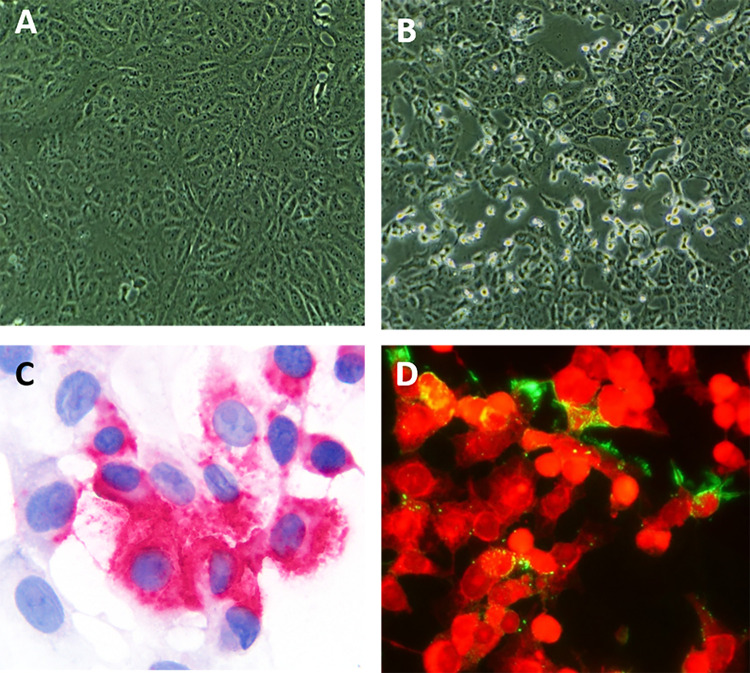
SARS-CoV-2 isolation from respiratory specimens from a tiger (Tiger 1). (A and B) Mock-infected control Vero cells (A) and Vero cells inoculated with tracheal wash fluid showing typical CPE at 48 h postinoculation (B). (C) ISH using a SARS-CoV-2 S-specific probe shows cytoplasmic red puncta. Red chromogenic assay with hematoxylin counterstain. (D) Immunofluorescence assay using a SARS-CoV N-specific monoclonal antibody shows SARS-CoV-2 replication in inoculated cells (green). Evans blue counterstain (red).

Virus isolation was performed on all respiratory and fecal samples. Cytopathic effect (CPE) was observed in Vero cells inoculated with tracheal wash fluid from Tiger 1 (Fig. 2A and B) and fecal samples from Tiger 3 and Lion 2 (Table S5). Results were confirmed by rRT-PCR (CDC N1 assay) and/or ISH and immunofluorescence assays (Fig. 2C and D). Additionally, a neutralizing antibody titer of 64 detected in Tiger 1 confirmed active SARS-CoV-2 infection in this animal (Table S6).

10.1128/mBio.02220-20.8 TABLE S5 Virus isolation for SARS-CoV-2 and rRT-PCR confirmation in tigers and lions. Download Table S5, DOCX file, 0.01 MB. Copyright © 2020 McAloose et al. 2020 McAloose et al. This content is distributed under the terms of the Creative Commons Attribution 4.0 International license.

10.1128/mBio.02220-20.9 TABLE S6 Virus neutralization assay on serum from Tiger 1. Download Table S6, DOCX file, 0.01 MB. Copyright © 2020 McAloose et al. 2020 McAloose et al. This content is distributed under the terms of the Creative Commons Attribution 4.0 International license.

### Epidemiologic and diagnostic investigation of zoo staff.

Subsequent to confirmation of SARS-CoV-2 infection in the animals, an epidemiologic investigation of zoo staff identified 10 zoo keepers and two managers who provided care for and had close (≤1.8-m) but not direct contact with the tigers or lions between 16 March 2020 (the date on which the zoo was closed to the public due to the pandemic) and 27 March to 3 April 2020 (timeline of disease onset in the animals). Four staff (2 tiger and 2 lion keepers) reported mild respiratory symptoms (including fever, cough, chills, myalgia, and fatigue) between 20 and 28 March 2020. Nasopharyngeal samples and blood were collected from these staff members on 6 April 2020, and rRT-PCR and a microsphere immunoassay (MIA; to detect IgG antibodies) were performed; staff who did not report symptoms were not tested. All tested keepers had evidence of current or prior SARS-CoV-2 infection (one rRT-PCR-positive tiger keeper [Keeper 1], one rRT-PCR- and serologically positive tiger keeper [Keeper 2], and two serologically positive lion keepers [Keepers 3 and 4]). All reported staying at home while sick. Whole-genome sequencing (WGS) of rRT-PCR-positive samples from Keepers 1 and 2 was performed to characterize the human samples and compare the human and animal viral genome sequences.

### Comparative genomics and phylogenetic and haplotype network analysis.

Nine complete SARS-CoV-2 genome sequences (from four tigers, three lions, and two keepers) and eight full-length S gene sequences (from seven symptomatic animals and one asymptomatic animal) were generated directly from respiratory and/or fecal samples (Data Sets 3 and 4). Compared to the Wuhan-Hu-1 sequence (GenBank accession number NC_045512), all tiger and keeper sequences contained six single-nucleotide polymorphisms (SNPs) with nine additional ambiguous sites (Fig. S2 and Table S7). A total of 20 sites differed between the three lion sequences and Wuhan-Hu-1 (Fig. S2 and Table S7).

10.1128/mBio.02220-20.2 FIG S2 SNP sites relative to Wuhan-Hu-1 genome. SNPs for tiger and keeper sequences are labeled along the top, and SNPs for lion sequences are labeled along the bottom. Blue bars indicate a unanimous consensus nucleotide across all assemblies for a given animal or keeper, and red bars indicate ambiguous base calls within that individual. Download FIG S2, TIF file, 0.6 MB. Copyright © 2020 McAloose et al. 2020 McAloose et al. This content is distributed under the terms of the Creative Commons Attribution 4.0 International license.

10.1128/mBio.02220-20.10 TABLE S7 Comparison of nucleotide and amino acid mutations between SARS-CoV-2 strains. Download Table S7, DOCX file, 0.1 MB. Copyright © 2020 McAloose et al. 2020 McAloose et al. This content is distributed under the terms of the Creative Commons Attribution 4.0 International license.

**FIG 3:**
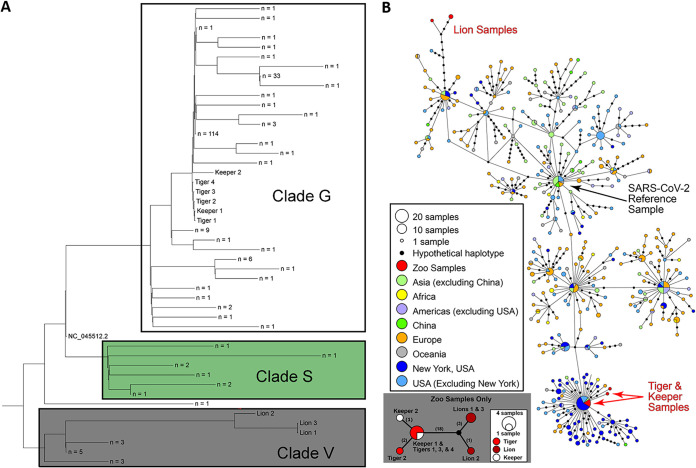
Phylogenetic and median-joining haplotype network analysis of SARS-CoV-2 strains in tigers, lions, and keepers. (A) Whole-genome phylogeny of zoo sequences with Wuhan-Hu-1 reference genome (NC_045512.2) and consensus sequences of other publicly available sequences from New York clustered at 99.99% identity (ML tree was unrooted and then midpoint rooted). (B) Haplotype analysis shows relatedness and levels of genetic variation between zoo and a global data set of SARS-CoV-2 genomes. Differences are indicated by one-step edges (lines) between black dots (hypothetical or unsampled haplotypes). Numbers in parentheses indicate differences between unique sequences (small panel; black dot = hypothetical sequence not sampled).

**FIG 4:**
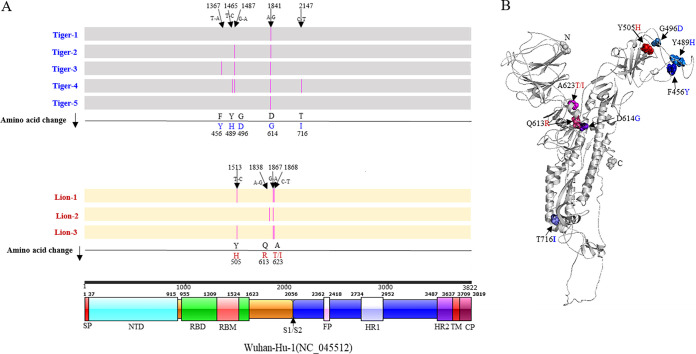
Nucleotide sequence and amino acid changes in the spike (S) protein of SARS-CoV-2 tigers and lions. (A) Comparison with Wuhan-Hu-1 (NC_045512). Nucleotide changes in each strain result in nonsynonymous substitutions (pink lines) that are listed in line with the amino acid change. Schematic representation of the organization and functional domains of the S protein for SARS-CoV2 Wuhan-Hu-1 (IBS online software). (B) Structural modeling of the SARS-CoV-2 spike protein. Homology modeling of the Tiger 1 spike protein (I-TASSER). S protein amino acid changes in the tiger and lion strains versus the Wuhan-Hu-1 strain are depicted in the structure.

Viral sequences in the tigers, lions, and keepers clustered into common SARS-CoV-2 clades (Fig. 3A). Those from tigers and tiger keepers clustered with clade G (defined by the D614G substitution in the spike protein); the lion sequences clustered with clade V (defined by the G251V substitution in ORF3a) (Fig. 3A). Median-joining haplotype network analysis of the viral sequences corroborated results of phylogenetic analyses (Fig. 3B and Data Set 4). Nucleotide sequence and amino acid analysis of the spike protein of SARS-CoV-2 in tigers and lions was performed. Compared with the Wuhan-Hu-1 strain, the tiger and lion SARS-CoV-2 S gene sequences had 1 to 4 nucleotide differences that resulted in several nonsynonymous substitutions (Fig. 4). Of five substitutions in the tiger strains, only one (G496D) was found in available human SARS-CoV-2 strains. These changes were not observed in the viral sequences from the lions (Fig. 4).

## DISCUSSION

Our results document susceptibility and natural SARS-CoV-2 infection in tigers and lions. These were the first confirmed animal infections in the United States and the first to be described in a nondomestic species in the world. Genomic and epidemiological data support a close evolutionary relationship between the viral strains in the tigers and those in the tiger keepers. Notably, the genetic differences and the distant phylogenetic relationship between sequences recovered from the tigers/tiger keepers and lions and the relationship of these strains in the context of global sequences indicate that tigers and lions were infected by two different SARS-CoV-2 genotypes. These data suggest that at least two independent SARS-CoV-2 introductions occurred, one in tigers and another in lions. Importantly, the SARS-CoV-2 genome sequence from Tiger 1 was identical to the viral sequence from Keeper 1 (a tiger keeper) and to other human SARS-CoV-2 strains detected in New York (NY-CDC-2929 [MT304486] and NY-QDX-00000001 [MT452574.1]). These observations, temporal overlap in animal and human infections, and a lack of new animal introductions to the collection support the conclusion of transmission from an infected keeper(s) to the tigers. Whether this was direct or indirect (e.g., fomite, food handling/preparation, or shared enrichment items) and whether subsequent tiger-to-tiger transmission (aerosol, respiratory droplet, etc.) occurred were not determined. A clear association and transmission source were not identified for the lions. The lion SARS-CoV-2 sequences were more divergent than those in the tigers and keepers. Interestingly, nine of the 12 SNPs (relative to the Wuhan-Hu-1 reference strain) shared by all three lion viruses were also found in a human strain (closest strain, GISAID accession no. EPI_ISL_427161) detected in Connecticut, USA. Two lion keepers were serologically positive for SARS-CoV-2, but viral RNA was not detected, and SARS-CoV-2 could not be confirmed in their respiratory samples. However, given close contact between keepers and animals and the serological evidence indicating infection of two of the lion keepers, it is possible that while asymptomatic, they or other asymptomatic staff may have transmitted virus to the lions.

The host range of SARS-CoV-2 and other coronaviruses is determined primarily by the interaction of the virus S glycoprotein, specifically the spike 1 subunit (S1), and the cellular receptor, angiotensin-converting enzyme II (ACE2) (19). *In silico* predictions have shown high binding potential between the S receptor binding domain (RBD) and domestic cat ACE2 receptor and conservation of three of five amino acid residues that are critical for interaction with the SARS-CoV-2 S glycoprotein in human and domestic cat ACE2 (19). These observations are supported by reports describing natural and experimental infection of domestic cats with SARS-CoV-2 (15, 20) and the data here that show a high degree of conservation between ACE2 in humans and domestic and wild felids. Further work is needed to determine if these changes affect SARS-CoV-2 receptor binding and pathogenicity in felids and humans.

Infections in the tigers and lions occurred at a time before SARS-CoV-2 testing was widely available in the United States and when there was limited evidence of pre- or asymptomatic viral shedding (21). Additionally, at that time, keepers caring for the tigers and lions did not generally wear personal protective equipment (PPE) given the (historical) low risk of infectious respiratory disease transmission between humans and domestic or nondomestic felid species. Results of this investigation prompted the immediate development of new protocols for PPE use in the enclosures of nondomestic felids and other known or susceptible species including mustelids, viverrids, and chiroptera (PPE was already in place for work with nonhuman primates) at the Bronx Zoo. They also contributed to the development of similar recommendations by other zoo and wildlife organizations (https://www.aza.org/aza-news-releases/posts/aza-and-aazv-statement-on-covid-19-positive-tiger-in-new-york).

The role of domestic and wild animal species in the epidemiology of SARS-CoV-2 is not completely understood. To date, the reported number of cases of SARS-CoV-2 infection in domestic and wild animal species is low, and to our knowledge, no other zoos worldwide have confirmed cases in their animals. This is notable when considered in the context of the large number of human cases and close interactions between people, their pets, and wild animals in their care. However, the fact that companion animals, farmed mink, and zoo animals are susceptible to SARS-CoV-2 infection and shed infectious virus in respiratory secretions and/or feces (13–15) makes the human-animal-environment interface an important area for further One Health-based studies (22). In general, a better understanding of SARS-CoV-2 susceptibility across a wide range of animal species will help to elucidate transmission mechanisms and identify potential reservoirs and sources of infection important in both animal and human health.

In the last 2 decades, at least three major coronavirus epidemics (SARS, MERS, and COVID-19) have occurred. A feature shared by these and other novel viruses of humans including ebolaviruses and human immunodeficiency virus (HIV) is an origin in a wild animal host. Despite a traditionally held perception of low risk, scientists and conservationists around the world have long recognized and shared concerns related to human activities that increase human-wildlife interactions and zoonotic disease transmission risk (22–24). As long as anthropogenic development and population growth bring humans and wildlife into increasing proximity, legal and illegal harvesting persists, and consumption of wildlife and wildlife products exists, there will be continued and significant risk of pandemic viral emergence with devastating global impact on human and animal health, economies, food security, and biodiversity.

## MATERIALS AND METHODS

### Sample collection.

Physical examination, thoracic and abdominal radiography and ultrasonography, and respiratory tract (nasal swab, oropharyngeal swab, and tracheal wash) and blood samples were collected on 2 April 2020 from an anesthetized female Malayan tiger (*Panthera tigris jacksoni*; Tiger 1) at the Wildlife Conservation Society’s (WCS’s) Bronx, Zoo, New York, NY, USA. The tiger developed an intermittent cough and wheezing on 27 March 2020. Additionally, voided fecal samples were collected opportunistically on 4 and 5 April 2020 from Tiger 1, all other tigers housed in the same facility as Tiger 1 (Malayan tiger [Tiger 2] and Amur tigers [*P. tigris altaica*; Tigers 3, 4, and 5]), and three African lions (*P. leo krugeri* [Lions 1, 2, and 3]) housed in a different facility within the zoo. All except Tiger 5 developed respiratory signs similar to those in Tiger 1 within a week (between 30 March and 3 April 2020) of disease onset in Tiger 1. Clinical signs resolved in less than 5 days (3 to 5 April 2020) in all animals except Tiger 1, whose clinical signs lasted for 16 days (resolution of clinical signs on 12 April 2020).

The tiger and lion facilities for animals in this study include internal housing areas and adjacent outdoor yards and exhibits. The tiger and lion facilities are located 457 m apart within the zoo. Tigers are housed individually in adjacent 42-m^2^ enclosures with three solid concrete walls, metal shift doors (to move animals between indoor and outdoor areas), and open-wire-mesh fronts. The animals are shifted between internal housing and one of two adjacent outdoor yards (144 m^2^ or 216 m^2^) or one of two outdoor natural environments (3,000 m^2^ each) through common spaces. Lions are housed individually in adjacent 9-m^2^ concrete enclosures with metal shift doors and a metal-mesh front. They are exhibited outdoors in alternating pairs in a shared, 1,081-m^2^ natural environment. No equipment was shared between the two facilities, and all delivery and preparation of food (the latter with gloved hands) were performed independently at the tiger and lion facilities.

Personal protective equipment (PPE), including N95 or surgical masks, face shields or goggles, and disposable gloves, were not generally worn when working with the tigers and lions prior to the SARS-CoV-2 pandemic but were worn during all animal handling and sample collection subsequent to the development of clinical signs in Tiger 1. Diagnostic specimens were submitted to the University of Illinois Veterinary Diagnostic Laboratory (UIUC-VDL) and Animal Health Diagnostic Center at Cornell University (Cornell AHDC) (both part of the National Animal Health Laboratory Network) for broad diagnostic investigation (respiratory samples—Tiger 1) and specific SARS-CoV-2 testing (fecal samples—all animals).

### Epidemiologic investigation.

Subsequent to the development of clinical signs and positive test results in the tigers and lions, an epidemiologic investigation into possible human infections in staff working with these animals was conducted by the New York City (NYC) and New York State (NYS) public health laboratories in conjunction with the U.S. Centers for Disease Control and Prevention (CDC). The investigation focused on the time period between 16 March 2020 (date on which the zoo was closed to the public due to the pandemic) and 27 March to 3 April 2020 (the timeline of disease onset in the animals). Twelve staff members (10 keepers and 2 managers) were identified who had responsibilities that offered opportunities for close (≤1.8-m) but not direct contact with the animals during this time period. This included moving animals between enclosures and exhibits, feeding, training sessions, enrichment activities, greetings, and social interactions (e.g., chuffing, a form of vocalization that tigers performed that involves air exhalation and which keepers also use to greet tigers). Additionally, lion keepers worked at a desk that was located less than 1.8 m from metal-mesh-fronted lion enclosures. Four keepers reported being mildly symptomatic (including fever, cough, chills, myalgia, and fatigue) with signs beginning in each prior to or concurrently with illness in animals on 20, 22, 27, and 28 March 2020. Nasopharyngeal swab and blood samples were collected on 6 April 2020 from the symptomatic keepers, and SARS-CoV-2-specific rRT-PCR and a microsphere immunoassay (to detect IgG antibodies) were performed; sampling and testing were not performed in the eight additional staff who did not report symptoms.

All four of the tested keepers had evidence of SARS-CoV-2 infection (one rRT-PCR-positive tiger keeper [Keeper 1], one rRT-PCR- and serology-positive tiger keeper [Tiger 2], and two serology-positive lion keepers [Keeper 3 and Keeper 4]). None of the keepers reported being sick at work. All stayed home for at least 7 days from the onset of illness, and none returned to work prior to a minimum of 7 symptom-free days and 72 fever-free hours in compliance with organizational COVID-19 policies and CDC and NY Department of Health (DOH) guidelines. rRT-PCR-positive specimens were forwarded to CDC for whole-genome sequencing (WGS) and haplotype network analysis to characterize the human samples and further compare the human and animal viral genome sequences. Interviews with the tiger and lion keepers suggested that up to two additional keepers may have had signs or symptoms suggestive of mild and transient COVID-19; however, they did not self-report being sick, may not have recognized their symptoms as being consistent with COVID-19, and were not tested.

### Non-SARS-CoV-2 respiratory pathogen testing.

Nucleic acid extracted from the respiratory specimens (nasal and oropharyngeal swabs and tracheal wash fluid) from Tiger 1 was tested by real-time PCR (rPCR) or rRT-PCR for several common feline respiratory pathogens (Cornell AHDC) including Bordetella bronchiseptica (25), influenza A virus (CDC universal assay) (26), Mycoplasma cynos (27), Mycoplasma felis (28), pneumovirus (29) with probe modification (6-carboxyfluorescein [FAM]-CTTCATCACTTTTGGCCTGGCCCAG-BHQ1), and Streptococcus equi subsp. *zooepidemicus* (30). Additionally, samples were tested for Chlamydia psittaci, Chlamydia felis, and Chlamydia abortus using a conventional PCR assay (31), and virus isolation was performed using inoculated feline pulmonary cells to test for feline herpesvirus and feline calicivirus. All assays have been adapted and optimized and are used routinely in feline infectious respiratory disease diagnostic testing.

### Tracheal wash fluid cytology.

Direct smears of buoyant, flocculent material in the tracheal wash fluid and cytocentrifuge smears of the remaining fluid were prepared and stained with a Romanoski stain (modified Wright’s stain) using an automated stainer (Hema-tek 1000; Siemens). The stained slides were examined using standard bright-field microscopy by a board-certified veterinary clinical pathologist (Cornell AHDC).

### *In situ* hybridization for SARS-CoV-2 RNA.

Unstained cytologic smears of tracheal wash fluid from Tiger 1 were fixed in ice-cold 100% methanol for 20 min and stored at −80°C until shipped to UIUC College of Veterinary Medicine Zoological Pathology Program (ZPP) on cold packs. Upon arrival, the slides were submerged for an additional 30 min in 10% neutral buffered formalin at 4°C, air dried, and then placed in 100% ethyl alcohol for 5 min at room temperature. An *in situ* hybridization (ISH) chromogenic manual assay was performed using the RNAscope 2.5 HD Detection Kit Red and a 20-pair oligonucleotide probe targeting the SARS-CoV-2 S gene of the Wuhan Hu-1 complete genome (NC_045512.2; Advanced Cell Diagnostics catalog no. 848561) according to the manufacturer’s directions (Advanced Cell Diagnostics, Inc., Newark, CA). Positive-control slides consisted of Vero cells infected with SARS-CoV-2 isolated from Tiger 1. A control probe targeting the DapB gene from the Bacillus subtilis strain SMY (Advanced Cell Diagnostics catalog no. 310043) was used as a negative control on all cytology sections in parallel with the SARS-CoV-2 target probe (see Fig. S1). Additional negative controls to rule out cross-reactivity with tiger RNA and the felid alphacoronavirus included a cytocentrifuge preparation of cell cultures infected with the alphacoronavirus feline enteric coronavirus (FeCoV) (kindly provided by Gary Whittaker, Cornell University); formalin-fixed, paraffin-embedded (FFPE) unstained sections of normal Malayan tiger trachea, lung, and oropharyngeal tissue; and lung, lymph node, and intestine from a FeCoV-positive domestic cat (Fig. S1). Samples from the control tiger and domestic cat were collected opportunistically in 2014 and 2013, respectively, and archived as part of routine necropsy procedures (Wildlife Conservation Society’s [WCS’s] Bronx Zoo and UIUC-ZPP, respectively).

### Virus isolation.

Virus isolation on respiratory and fecal samples was performed in Vero (ATCC CCL-81), Vero E6, and Vero 76 cells under biosafety level 3 conditions at the Cornell AHDC and the National Veterinary Services Laboratory (NVSL). Cells were cultured in minimum essential medium Eagle (MEM-E; Gibco, Gaithersburg, MD) supplemented with 2.5 to 10% fetal bovine serum (FBS; Gibco), 100 IU/ml penicillin, and 100 μg/ml streptomycin (growth medium). Cells were seeded in 12-well culture plates or T25 flasks and cultured at 37°C with 5% CO_2_ for 24 to 48h. Before inoculation, respiratory swabs and tracheal wash fluid samples were diluted at 1:10, 1:100, and 1:1,000 in serum-free MEM-E containing 200 UI/ml penicillin, 200 μg/ml streptomycin, and 2.5 μg/ml amphotericin B (all from Gibco); swabs from fecal samples were placed in 1 ml of sterile PBS supplemented with 2.5% bovine serum albumin (BSA) (Sigma-Aldrich, St. Louis, MO) containing 200 UI/ml penicillin, 200 μg/ml streptomycin, and 2.5 μg/ml amphotericin B (all from Gibco). Cells were rinsed with MEM-E and inoculated with 300 μl of each respiratory sample dilution in individual wells of a 12-well plate or 1.5 ml of the diluted fecal sample in a T25 flask and adsorbed for 1 h at 37°C with 5% CO_2_ for 1 h. Mock-inoculated cells were used as negative controls. After adsorption, replacement medium was added, and cells were incubated at 37°C with 5% CO_2_ and monitored daily for cytopathic effect (CPE) for 5 days. Cell cultures with no CPE were frozen, thawed, and subjected to three blind passages with inoculation of fresh Vero cell cultures with the lysates as described above. SARS-CoV-2 infection in CPE-positive cultures was confirmed with SARS-CoV-2-specific rRT-PCR using the CDC N1 primer and probe set (sequences available upon request), an immunofluorescence assay using a mouse monoclonal antibody against the SARS-CoV N protein (32, 33), and RNAscope *in situ* hybridization as described above.

### Virus neutralization assay.

Seroconversion of Tiger 1 to SARS-CoV-2 was assessed by a virus neutralization assay (VN; Cornell AHDC). Twofold serial dilutions (1:8 to 1:4,096) of a serum sample collected on 2 April 2020 (6 days after the onset of clinical signs) were incubated with 100 50% tissue culture infective doses (TCID_50_) of SARS-CoV-2 on Vero cells for 1 h at 37°C. Following incubation of serum and virus, 50 μl of a cell suspension of Vero CCL-81 cells was added to each well of a 96-well plate and incubated for 72 h at 37°C with 5% CO_2_. Virus cytopathic effect (CPE) was used as an indicator of virus infection/replication. Neutralizing antibody titers were expressed as the reciprocal of the highest dilution of serum that completely inhibited CPE. Archived frozen sera from another tiger (archived frozen at Cornell AHDC) and positive human control sera (deidentified convalescent human sera provided by Cayuga Medical Center, IRB protocol 0420EP) were included, and all samples were tested in triplicate with results averaged. A cell culture control was included in the assays, and the virus working dilution was back-titrated.

### SARS-CoV-2 rRT-PCR.

Nucleic acid was extracted from nasal and oropharyngeal swabs and tracheal wash fluid (Tiger 1) using the MagMAX isolation kit (Thermo Fisher Scientific, Waltham, MA) and from fecal samples (Tigers 1 to 5 and Lions 1 to 3) using the MagMAX Core nucleic acid purification kit (Thermo Fisher Scientific, Waltham, MA) and an automated nucleic acid extractor (King Fisher flex purification system; Thermo Fisher Scientific, Waltham, MA) according to the manufacturer’s instructions. Analysis for SARS-CoV-2 RNA was performed with real-time reverse transcriptase (rRT) PCR and either the 2019-nCoV CDC qPCR probe assay targeting three regions of the nucleocapsid (N) gene (N1, N2, and/or N3; Integrated DNA Technologies [IDT], Inc., Coralville, IA) (UIUC-VDL, Cornell AHDC) or the CDC qPCR probe assay, and in-house primers for the envelope (E) gene with the AgPath-ID one-step RT-PCR kit (Applied Biosystems, Foster City, CA) (UIUC-VDL) (primer and probe sequences are available upon request). Thermal cycler conditions consisted of reverse transcription and enzyme activation at 45 to 48°C for 10 min and 95°C for 10 min, respectively, followed by 40 to 45 cycles of 95°C for 3 to 15 s and 55 to 60°C for 45 s. Positive (2019-nCoV_N_Positive Control; IDT, Coralville, IA, and a synthesized plasmid [GenScript] E gene control) and negative (distilled or nuclease-free water) controls, plus internal amplification controls (Xeno or beta-actin; Thermo Fisher Scientific, Waltham, MA), were included as separate reaction mixtures. Following initial rRT-PCR testing at both institutions, samples were submitted to the NVSL for confirmatory testing, using the 2019-nCoV CDC qPCR probe assay and N1 and N2 primers (sequences available upon request).

### Amplicon sequencing.

MinION and Sanger amplicon sequencing was used to confirm rRT-PCR results at Cornell AHDC and NVSL, respectively (primer sequences are available upon request). For MinION-based sequencing, targets were amplified directly from tracheal wash or fecal samples using the SuperScript IV one-step RT-PCR system (Thermo Fisher Scientific, Waltham, MA). Primers targeted the complete spike (S) gene (4,023 bp) and an internal region of the N gene (634 bp). Universal Oxford Nanopore-compatible adapter sequences were added to the 5′ end of each primer sequence to allow PCR-based barcoding. Amplicons were purified (AMPure XP beads [Beckman Coulter, Brea, CA]; 1.6:1 volumetric bead-to-DNA ratio), and DNA quantification was performed on a Qubit fluorometer 3.0 (double-stranded DNA [dsDNA] high-sensitivity assay kit; Thermo Fisher Scientific, Waltham, MA). Samples were subsequently diluted to 0.5 nM in a total of 24 μl and used as the input for the library preparation following the 1D PCR barcoding (96) genomic DNA (SQK-LSK109) protocol (Oxford Nanopore Technologies, Oxford, UK). Final DNA libraries were loaded in a Flo-MIN106 R9.4 flow cell to start the sequencing runs.

Sanger sequencing was performed using primers targeting partial regions of S, N, and RNA-dependent RNA polymerase (RdRp) genes (primer sequences available upon request). Amplicons were generated directly from nasal and oropharyngeal swabs and tracheal wash fluid using the SuperScript III one-step RT-PCR System (Thermo Fisher Scientific, Waltham, MA). Reaction mixtures were purified using the Qiagen PCR purification kit (Qiagen, Germantown, MD). DNA was amplified for Sanger sequencing using the BigDye Terminator v 3.1 cycle sequencing kit (Thermo Fisher Scientific, Waltham, MA) and sequenced on the Applied Biosystems 3500xl genetic analyzer (Thermo Fisher Scientific, Waltham, MA).

### Whole-genome sequencing and phylogenetic analysis.

Whole-genome sequencing (WGS) was performed on tracheal wash fluid and fecal specimens from all individual tigers and lions as previously described (34). Individual fecal samples from Tigers 1 to 5 and Lions 1 to 3 and cell culture viral isolates were subjected to sequencing with either MinION-based amplicon sequencing using overlapping primers covering the full viral genome (amplicons with an average size of ∼1,500 bp; primer sequences are available upon request) or the Ion AmpliSeq kit for Chef DL8 and Ion AmpliSeq SARS-CoV-2 research panel (Thermo Fisher Scientific, Waltham, MA) (Data Sets 2 and 3). MinION libraries were prepared as previously described (35) using the Native Barcode kit, EXP-NBD104, ligation sequencing kit, SQK-SQK109 (Oxford Nanopore Technologies), and sequenced on an R9.4 flow cell for 6 h. Ion targeted libraries were sequenced using an Ion 530 chip on the Ion S5 system using the Ion 510-Ion 520-Ion 530 kit (Thermo Fisher Scientific, Waltham, MA). Viral isolates were sequenced with the MinION or Ion AmpliSeq approach as described above. Whole-genome sequencing on the rRT-PCR-positive specimens from the two SARS-CoV-2-positive keepers was performed as previously described using an amplicon sequencing approach and Sanger sequencing (36) (Data Set 4).

All genomes for each animal that were assembled using data generated from different sequencing platforms (Illumina, MinION, and/or Ion Torrent) were combined into a single consensus sequence for each animal (Data Set 2). The assemblies for a given animal were aligned with the Wuhan-Hu-1 reference sequence (NC_045512.2) using MAFFT v. 7.453 (37). The reference sequence was then removed from the alignment, and a consensus sequence for the virus sequence recovered from each animal was generated using the consambig program in EMBOSS v. 6.6.0.0 (38). When a single assembly shifted alignment due to a single base insertion or repeat nucleotide, the alignment was rerun after removal of the offending nucleotides.

To compare the outbreak genomes to others isolated from humans in the same geographic region, all available SARS-CoV-2 genomes from New York were downloaded from NCBI on 23 April 2020. These were clustered at 99.99% identity using vsearch v. 2.14.2 (39), and the consensus sequences from each cluster were aligned along with Wuhan-Hu-1 reference sequence, using MAFFT v. 7.453 (37). A phylogenetic tree was constructed using the consensus sequence from each animal, keepers, sequences from New York, and the Wuhan-Hu-1 reference sequence (NC_045512.2) using the GTR-Gamma model in RAxML v. 8.2.12 (40).

### Haplotype network analysis.

Haplotype network analyses were conducted with two overlapping data sets using PopART software (41) using the median joining algorithm (42). Data Set 1 contained nine genomes generated from the Bronx Zoo cases: four tigers, three lions, and two tiger keepers. Data Set 2 contained the nine genomes from Data Set 1, as well as 500 additional genomes (including the SARS-CoV-2 reference Wuhan-Hu genome) generated from a total of 53 countries to better understand genetic relatedness of Bronx Zoo cases in the context of the global pandemic. The top 10 BLAST results for the lion sequences were included in Data Set 2.

For both data sets, the entire genome alignment was examined visually for accuracy and evidence of large-scale rearrangements to rule out the likelihood of multiple single-nucleotide polymorphisms (SNPs) being the result of a single evolutionary event. Subsequently, whole-genome alignments were converted into an SNP matrix by removing columns containing identical bases, gaps, and ambiguous bases. The lengths of final SNP matrices were 25 nucleotides (nt) (Data Set 1) and 567 nt (Data Set 2).

### Data availability.

Primer and probe sequence information for rRT-PCR and amplicon and whole-genome sequencing is available upon request. All remaining data are available in the main text or the supplemental material or as follows: Data Set 1, metagenomics data obtained from respiratory specimens from Tiger 1 have been deposited in SRR11587605 under PRJNA627354; Data Set 2, whole-genome consensus sequences obtained from SARS-CoV-2 detected in fecal samples from Tigers 2 to 4 and Lions 1 to 3 have been deposited in GenBank under accession numbers MT704313, MT704315, MT704316, MT704312, MT704310, and MT704311, respectively; Data Set 3, whole-genome sequences obtained from SARS-CoV-2 tiger and lion isolates (TGR1/NY/20, TGR1/NY/20, and LN2/NY/20) have been deposited in GenBank under accession numbers MT704317, MT704314, and MT747978, respectively; Data Set 4, whole-genome sequences obtained from SARS-CoV-2 strains in Keepers 1 and 2 have been deposited in GenBank under accession numbers MT703883 and MT703884, respectively.

10.1128/mBio.02220-20.3 FIG S3 Alignment of human, domestic cat, and tiger in ACE2 amino acid sequences. Amino acid alignment was performed using MAFFT with parameters Auto for algorithm, BLOSUM62 for scoring matrix, Gap open penalty of 1.53, and Offset value of 0.123. Positions where amino acid differences were noted are highlighted in colors. Download FIG S3, PPT file, 2.0 MB. Copyright © 2020 McAloose et al. 2020 McAloose et al. This content is distributed under the terms of the Creative Commons Attribution 4.0 International license.
