# Supplementary figures and images for: From People to Panthera: Natural SARS-CoV-2 Infection in Tigers and Lions at the Bronx Zoo
Source: mBio. 2020 Oct 13;11(5):e02220-20. doi: 10.1128/mBio.02220-20 (PMC7554670; doi:10.1128/mBio.02220-20)

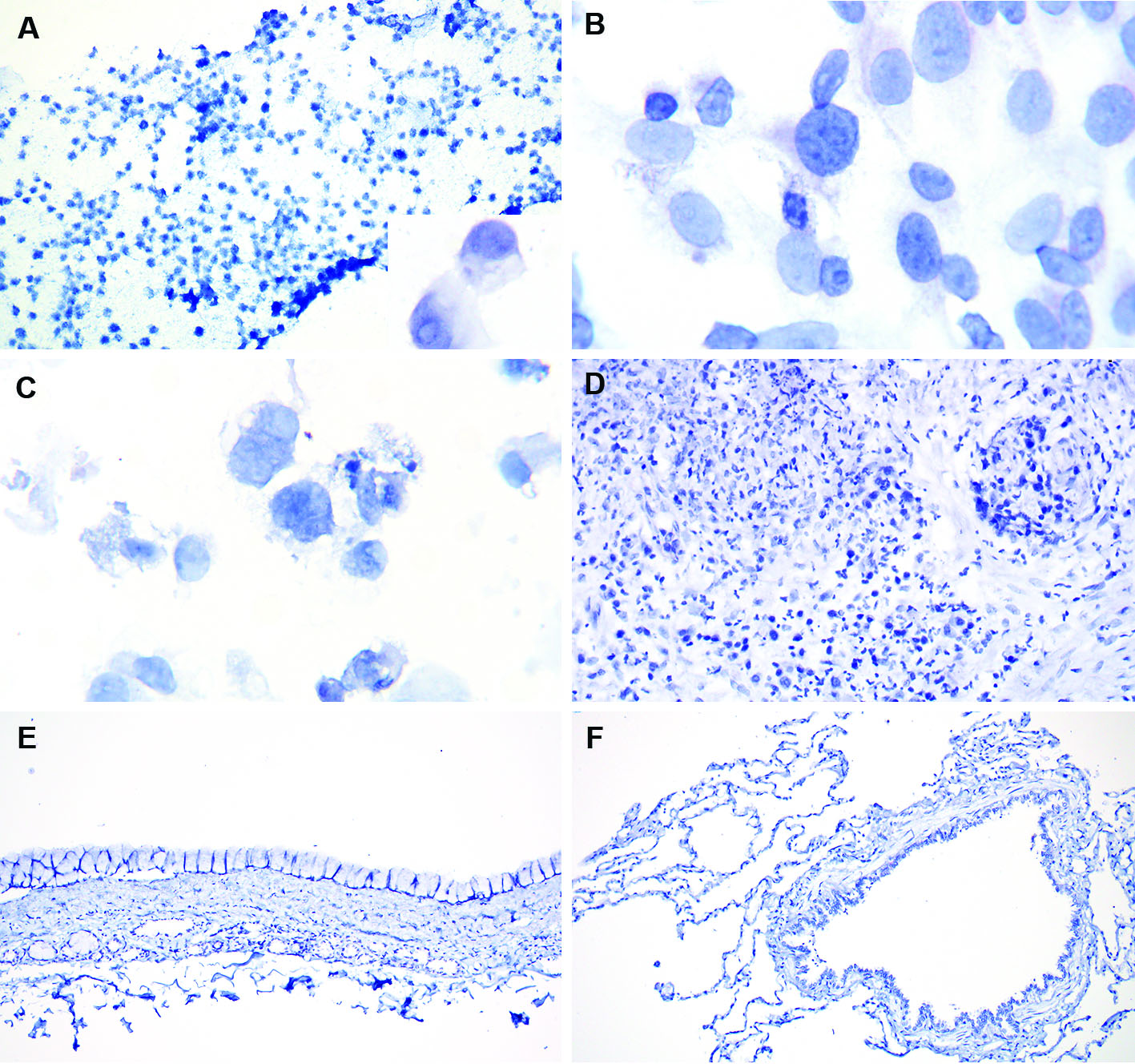

Supplement: FIG S1 [file mBio.02220-20-sf001.jpg]

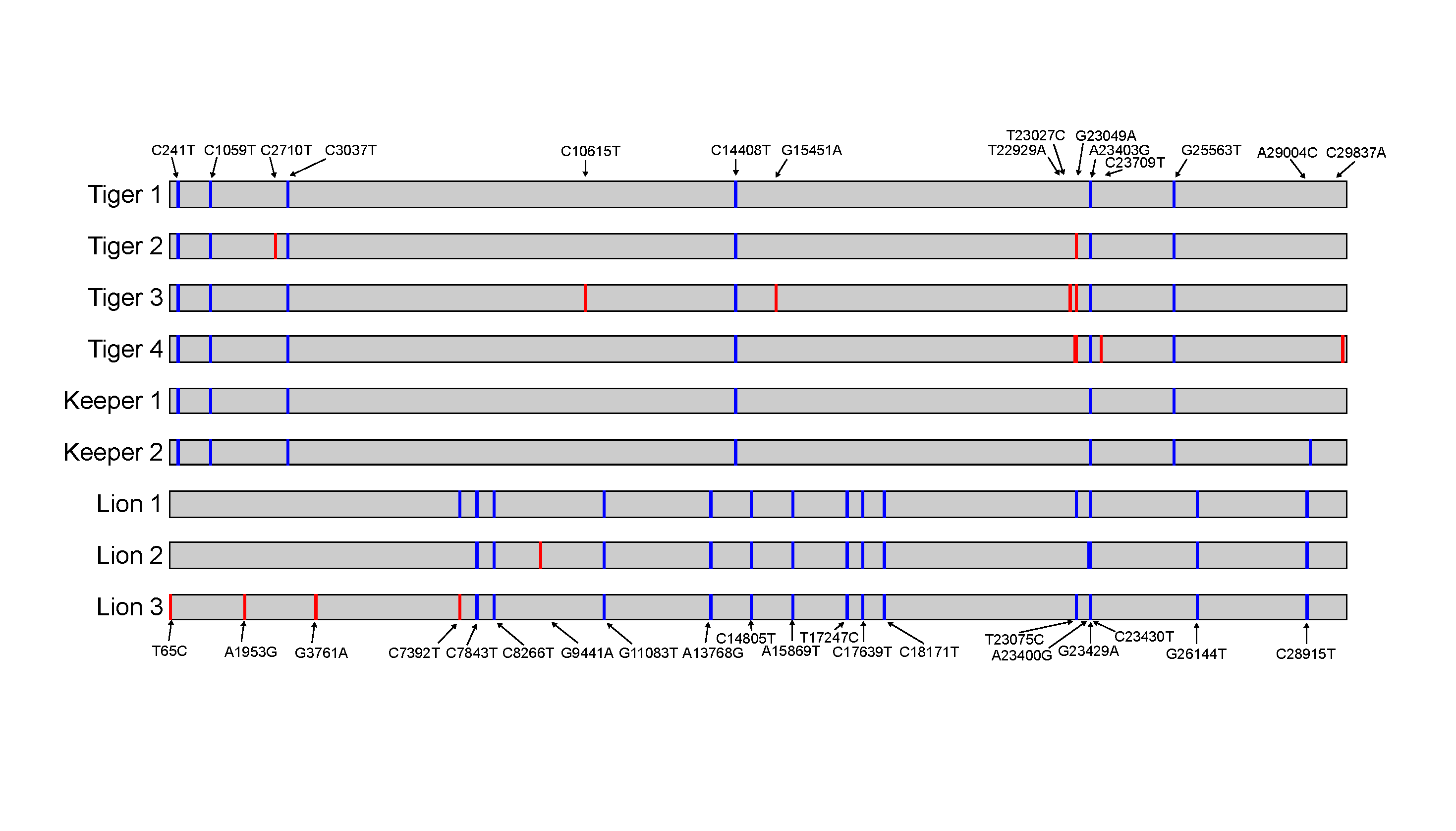

Supplement: FIG S2 [file mBio.02220-20-sf002.tif]

## Slide 1
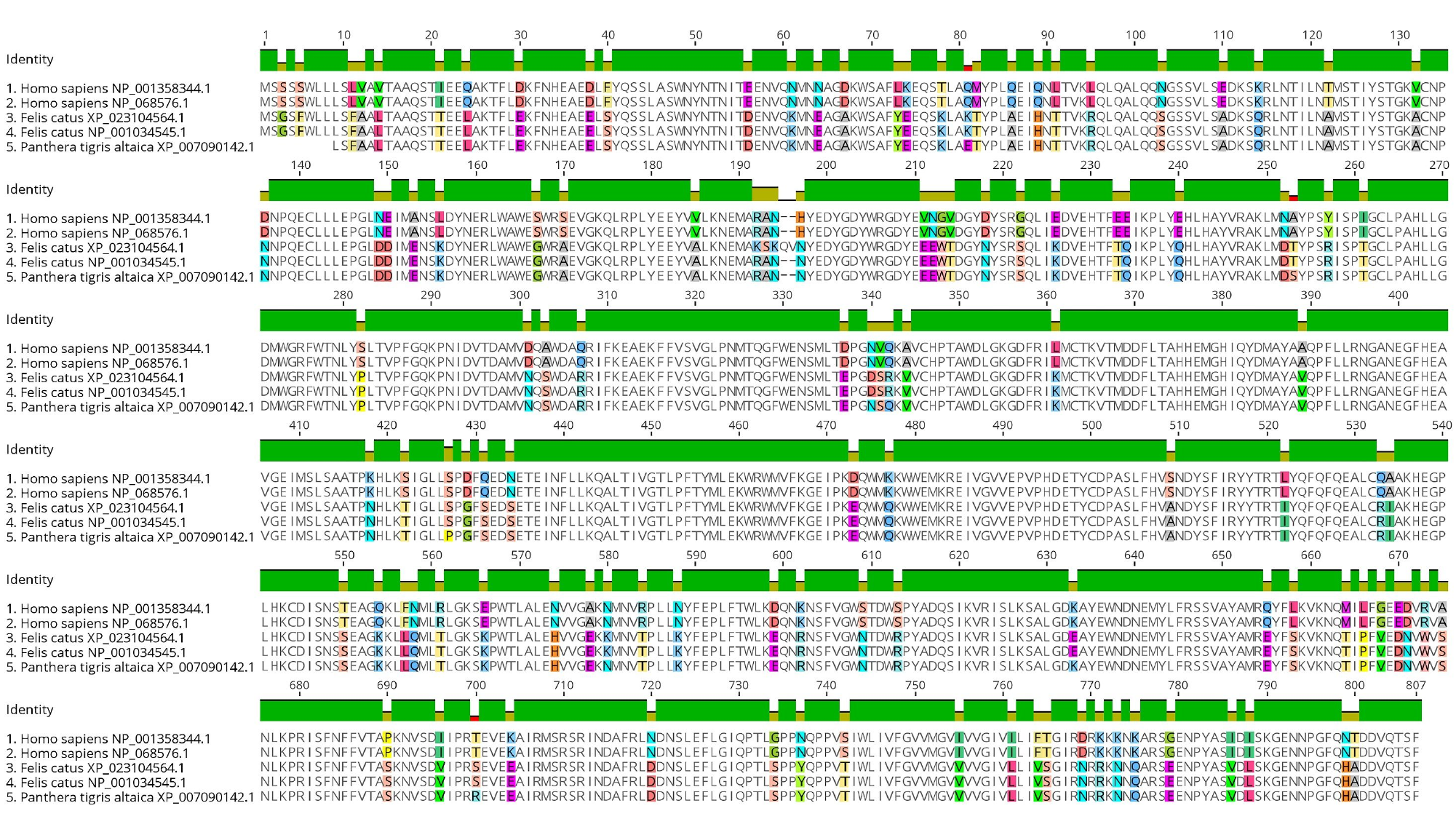

Supplement: FIG S3 [file mBio.02220-20-sf003.ppt]
