# Supplementary material for: From People to Panthera: Natural SARS-CoV-2 Infection in Tigers and Lions at the Bronx Zoo
Source: mBio. 2020 Oct 13;11(5):e02220-20. doi: 10.1128/mBio.02220-20 (PMC7554670; doi:10.1128/mBio.02220-20)
Supplement: TABLE S1 [file mBio.02220-20-st001.docx]

**Table S1. Targeted feline respiratory pathogen testing in Tiger 1.**

| PCR target | Specimen | Result | Specimen | Result |
| --- | --- | --- | --- | --- |
| Influenza A virus | Tracheal wash | Not detected | NS/OP | Not detected |
| Pneumovirus | Tracheal wash | Not detected | NS/OP | Not detected |
| *Mycoplasma cynos* | Tracheal wash | Not detected | NS/OP | Not detected |
| *Mycoplasma felis* | Tracheal wash | Not detected | NS/OP | Not detected |
| *Bordetella* spp. | Tracheal wash | Not detected | NS/OP | Not detected |
| *Streptococcus zooepidemicus* | Tracheal wash | Not detected | NS/OP | Not detected |
| *Chlamydia* spp.^*^ | Tracheal wash | Not detected | NS/OP | Not detected |
| Virus isolation^†^ | Specimen | Result | Specimen | Result |
| Feline herpesvirus | Tracheal wash | No virus isolated | NS/OP | No virus isolated |
| Feline calicivirus | Tracheal wash | No virus isolated | NS/OP | No virus isolated |

^*^This PCR detects *C. psittaci*, *C. felis* and *C. abortus*.

^†^Virus isolation was performed in feline lung cells.

NS = nasal swab sample; OP = oropharyngeal swab sample.
