# Supplementary material for: From People to Panthera: Natural SARS-CoV-2 Infection in Tigers and Lions at the Bronx Zoo
Source: mBio. 2020 Oct 13;11(5):e02220-20. doi: 10.1128/mBio.02220-20 (PMC7554670; doi:10.1128/mBio.02220-20)
Supplement: TABLE S2 [file mBio.02220-20-st002.docx]

**Table S2. Average rRT-PCR Ct values for SARS-CoV-2 targets in respiratory samples.**

|  | Cornell AHDC | |  | UIUC_VDL | | | NVSL | | |
| --- | --- | --- | --- | --- | --- | --- | --- | --- | --- |
| Tiger 1 Sample/Target | N1 | N2 | N3 | N2 | E |  | N1 | N2 | |
| Nasal | 21.26^*^ | 20.99 | 20.59 | 19.94 | 28.13 |  | 18.20 | | 18.30 |
| Oropharyngeal |  |  |  | 28.23 | 35.00 |  | 27.00 | | 27.60 |
| Tracheal wash 1 | 20.97^†^ | 21.00 | 20.46 | 21.49 | 29.01 |  | 20.05 | | 20.35 |
| Tracheal wash 2 |  |  |  | 21.73 | 28.71 |  | 20.25 | | 20.10 |

^*^Pooled nasal and oropharyngeal swab samples were tested.

^†^Pooled aliquots from a single tracheal wash fluid sample were tested.

UIUC-VDL, Cornell AHDC Ct cutoff values: N1, N2, N3 < 40; E < 37

NVSL Ct cutoff values: N1, N2 < 40.
