# Supplementary material for: From People to Panthera: Natural SARS-CoV-2 Infection in Tigers and Lions at the Bronx Zoo
Source: mBio. 2020 Oct 13;11(5):e02220-20. doi: 10.1128/mBio.02220-20 (PMC7554670; doi:10.1128/mBio.02220-20)
Supplement: TABLE S3 [file mBio.02220-20-st003.docx]

**Table S3. Targeted MinION amplicon sequencing in tracheal wash fluid and fecal samples.**

| Full SARS-CoV-2 S gene | | | |  |  |
| --- | --- | --- | --- | --- | --- |
|  | N. of reads^*^ | Mean depth of coverage | Minutes of sequencing^†^ | BLAST search | Percent id. |
| Tiger 1^‡^ | 200 | 141.3 | 1 | [LC547533.1](https://www.ncbi.nlm.nih.gov/nucleotide/LC547533.1?report=genbank&log$=nucltop&blast_rank=1&RID=CND1GJJH014) | 100 |
| Tiger 2^§^ | 1066 | 1052.9 | 30 | [LC547533.1](https://www.ncbi.nlm.nih.gov/nucleotide/LC547533.1?report=genbank&log$=nucltop&blast_rank=1&RID=CNDFNSCA014) | 99.98 |
| Tiger 3^§^ | 2405 | 2478.3 | 30 | [LC547533.1](https://www.ncbi.nlm.nih.gov/nucleotide/LC547533.1?report=genbank&log$=nucltop&blast_rank=1&RID=CNDFNSCA014) | 99.98 |
| Tiger 4^§^ | 2921 | 2892.5 | 30 | [LC547533.1](https://www.ncbi.nlm.nih.gov/nucleotide/LC547533.1?report=genbank&log$=nucltop&blast_rank=1&RID=CNDFNSCA014) | 99.95 |
| Tiger 5^§^ | 44 | 22.1 | 150 | [LC547533.1](https://www.ncbi.nlm.nih.gov/nucleotide/LC547533.1?report=genbank&log$=nucltop&blast_rank=1&RID=CNDX7GC2016) | 100 |
| Lion 1^§^ | 30 | 14.8 | 150 | [MT447170.1](https://www.ncbi.nlm.nih.gov/nucleotide/MT447170.1?report=genbank&log$=nucltop&blast_rank=1&RID=CNE1XF5A014) | 100 |
| Lion 2^§^ | 2432 | 2389.8 | 30 | [MT447170.1](https://www.ncbi.nlm.nih.gov/nucleotide/MT447170.1?report=genbank&log$=nucltop&blast_rank=1&RID=CNE4GXYE016) | 99.95 |
| Lion 3^§^ | 77 | 27.2 | 150 | [LC547533.1](https://www.ncbi.nlm.nih.gov/nucleotide/LC547533.1?report=genbank&log$=nucltop&blast_rank=1&RID=CNEBAHET01R) | 100 |
|  |  |  |  |  |  |
| Partial SARS-CoV-2 N gene | | | |  |  |
|  | N. of reads | Mean depth of coverage | Minutes of sequencing | BLAST search | Percent id. |
| Tiger 1^‡^ | 482 | 458.8 | 1 | [LC547522.1](https://www.ncbi.nlm.nih.gov/nucleotide/LC547522.1?report=genbank&log$=nucltop&blast_rank=1&RID=CND7ZZDS014) | 100 |
| Tiger 2^§^ | 16323 | 16126.7 | 30 | [LC547522.1](https://www.ncbi.nlm.nih.gov/nucleotide/LC547522.1?report=genbank&log$=nucltop&blast_rank=1&RID=CNEE06PV014) | 100 |
| Tiger 3^§^ | 23455 | 23255.8 | 30 | [LC547522.1](https://www.ncbi.nlm.nih.gov/nucleotide/LC547522.1?report=genbank&log$=nucltop&blast_rank=1&RID=CNEE06PV014) | 100 |
| Tiger 4^§^ | 20141 | 19934.9 | 30 | [LC547522.1](https://www.ncbi.nlm.nih.gov/nucleotide/LC547522.1?report=genbank&log$=nucltop&blast_rank=1&RID=CNEE06PV014) | 100 |
| Tiger 5^§^ | 1479 | 1459.7 | 30 | [LC547522.1](https://www.ncbi.nlm.nih.gov/nucleotide/LC547522.1?report=genbank&log$=nucltop&blast_rank=1&RID=CNEE06PV014) | 100 |
| Lion 1^§^ | 109 | 106.8 | 30 | [MT358693.1](https://www.ncbi.nlm.nih.gov/nucleotide/MT358693.1?report=genbank&log$=nucltop&blast_rank=1&RID=CP0317D501R) | 99.84 |
| Lion 2^§^ | 17513 | 17360.3 | 30 | [MT358693.1](https://www.ncbi.nlm.nih.gov/nucleotide/MT358693.1?report=genbank&log$=nucltop&blast_rank=1&RID=CP05R17Y014) | 100 |
| Lion 3^§^ | 1290 | 1273 | 30 | [MT358693.1](https://www.ncbi.nlm.nih.gov/nucleotide/MT358693.1?report=genbank&log$=nucltop&blast_rank=1&RID=CP07R6NR014) | 100 |

^*^ Number of reads mapped to genome used as reference ([MN985325.1](https://www.ncbi.nlm.nih.gov/nucleotide/MN985325.1?report=genbank&log$=nucltop&blast_rank=1&RID=997BCEHE016)) after processing raw reads.

^†^ Minutes necessary after starting sequencing run to generate consensus sequence.

^‡^Tracheal wash fluid sample.

^§^Fecal sample.
