# Supplementary material for: From People to Panthera: Natural SARS-CoV-2 Infection in Tigers and Lions at the Bronx Zoo
Source: mBio. 2020 Oct 13;11(5):e02220-20. doi: 10.1128/mBio.02220-20 (PMC7554670; doi:10.1128/mBio.02220-20)
Supplement: TABLE S4 [file mBio.02220-20-st004.docx]

**Table S4. Average rRT-PCR Ct values for SARS-CoV-2 targets in fecal samples.**

| Laboratory | Cornell AHDC | Cornell AHDC | Cornell AHDC | UIUC- VDL | UIUC- VDL |
| --- | --- | --- | --- | --- | --- |
| Sample Collection Date | 4-Apr-20 | 4-Apr-20 | 4-Apr-20 | 5-Apr-20 | 5-Apr-20 |
| Animal ID/rRT-PCR | N1 | N2 | N3 | N2 | E |
| Tiger 1 | NA | NA | NA | 23.32 | 33.54 |
| Tiger 2 | 22.28 | 25.83 | 24.18 | 14.48 | 26.08 |
| Tiger 3 | 24.56 | 29.51 | 26.18 | 27.37 | 34.00 |
| Tiger 4 | 27.91 | 31.72 | 29.25 | 24.67 | 35.22 |
| Tiger 5 | 32.03 | 36.28 | 32.87 | 28.84 | 0 |
| Lion 1 | 31.04 | 35.69 | 33.51 | 25.13 | 33.64 |
| Lion 2 | 23.19 | 26.17 | 23.84 | 17.29 | 28.63 |
| Lion 3 | 31.22 | 34.83 | 34.10 | 24.97 | 35.00 |

NA = sample not available.

Ct cutoff values: N1, N2, N3 < 40; E < 37
