# Supplementary material for: From People to Panthera: Natural SARS-CoV-2 Infection in Tigers and Lions at the Bronx Zoo
Source: mBio. 2020 Oct 13;11(5):e02220-20. doi: 10.1128/mBio.02220-20 (PMC7554670; doi:10.1128/mBio.02220-20)
Supplement: TABLE S5 [file mBio.02220-20-st005.docx]

**Table S5. Virus isolation for SARS-CoV-2 and rRT-PCR confirmation in tigers and lions.**

| Laboratory | Sample | Cornell AHDC | | | NVSL | | | Cornell AHDC | | |
| --- | --- | --- | --- | --- | --- | --- | --- | --- | --- | --- |
| Sample Collection Date |  | 4-Apr-20 | | | 4-Apr-20 | | | 8-Apr-20 | | |
| Animal ID/Passage (P) |  | P 1 | P 2 | P 3 | P 1 | P 2 | P 3 | P 1 | P 2 | P 3 |
| Tiger 1 | Tracheal wash | - | + | + | - | - | - | NA | NA | NA |
| Tiger 1 | Nasal/oropharyngeal swab | - | - | - | - | - | - | NA | NA | NA |
| Tiger 2 | Feces | - | - | - | - | - | - | - | - | - |
| Tiger 3 | Feces | - | - | - | - | - | - | - | - | + |
| Tiger 4 | Feces | - | - | - | - | - | - | - | - | - |
| Tiger 5 | Feces | - | - | - | - | - | - | - | - | - |
| Lion 1 | Feces | - | - | - | - | - | - | - | - | - |
| Lion 2 | Feces | - | - | - | - | + | + | - | - | - |
| Lion 3 | Feces | - | - | - | - | - | - | - | - | - |

NA = sample not available.

-: No virus isolated; +: SARS-CoV-2 isolated, with confirmation by rRT-PCR (all positive samples), immunofluorescence staining for the N protein, and *in situ* hybridization (Tiger 1, tracheal wash).
