# Supplementary material for: From People to Panthera: Natural SARS-CoV-2 Infection in Tigers and Lions at the Bronx Zoo
Source: mBio. 2020 Oct 13;11(5):e02220-20. doi: 10.1128/mBio.02220-20 (PMC7554670; doi:10.1128/mBio.02220-20)
Supplement: TABLE S6 [file mBio.02220-20-st006.docx]

Table S6. Virus neutralization assay on serum from Tiger 1^*^.

| Sample^†,‡^ | 1:4 | 1:8 | 1:16 | 1:32 | 1:64 | 1:128 | 1:256 | 1:512 | 1:1028 | 1:2048 |
| --- | --- | --- | --- | --- | --- | --- | --- | --- | --- | --- |
| Negative control^§^ | + | + | + | + | + | + | + | + | + | + |
| Positive control^\|\|^ | - | - | - | - | - | - | + | + | + | + |
| Tiger 1 | - | - | - | - | - | + | + | + | + | + |

^*^Sample collection on April 2, 2020 (six days after respiratory signs were noted).

^†^Serial dilutions were incubated with 100 TCID_50_ of SARS-CoV-2 isolate TGR/NY/20 on Vero cells. Virus cytopathic effect was used as an indicator of virus infection/replication.

^‡^Antibody titers were defined as the reciprocal of the highest serum dilution that completely inhibited CPE (1:64 for Tiger 1).

^§^ Serum from another tiger collected in 2019 and available in Cornell AHDC frozen archives.

^||^ Convalescent human serum (IRB #0420EP).

-: No CPE (inhibition of infection/replication)

+: Presence of CPE.
