# Supplementary material for: From People to Panthera: Natural SARS-CoV-2 Infection in Tigers and Lions at the Bronx Zoo
Source: mBio. 2020 Oct 13;11(5):e02220-20. doi: 10.1128/mBio.02220-20 (PMC7554670; doi:10.1128/mBio.02220-20)
Supplement: TABLE S7 [file mBio.02220-20-st007.docx]

Table S7. Comparison of nucleotide and amino acid mutations between SARS-CoV-2 strains^*^.

| **Position** | **Subject** | **Nucleotide Change**^†^ | **Amino Acid Change**^‡^ | **Gene** |
| --- | --- | --- | --- | --- |
| 241 | All | C / T | NA | 5’UTR |
| 1059 | All | C / T | T / I | Nsp2 |
| 2710 | Tiger 2 | C / Y | No change | Nsp2 |
| 3037 | All | C / T | No change | Nsp3 |
| 10615 | Tiger 3 | C / Y | No change | 3C-like proteinase |
| 14408 | All | C / T | P / L | Nsp12 (RdRp) |
| 15451 | Tiger 3 | G / R | G / S | Nsp12 (RdRp) |
| 22929 | Tiger 3 | T / W | F / Y | S |
| 23027 | Tiger 4 | T / Y | Y / H | S |
| 23049 | Tiger 2  Tiger 3  Tiger 4 | G / R | G / D | S |
| 23403 | All | A / G | D / G | S |
| 23709 | Tiger 4 | C / Y | T / I | S |
| 25563 | All | G / T | Q / H | ORF3a |
| 29004 | Keeper 2 | A / C | Q / P | N |
| 29837 | Tiger 4 | C / M | NA | 3’UTR |

^*^Comparisons of SARS-CoV-2 sequences from five tigers, three lions, two keepers (Keeper 1 and 2) and the Wuhan-Hu-1 strain across the entire SARS-CoV-2 genome.

^†^For ambiguous base calls, the following ambiguity codes were used: Y = C or T; R = A or G; M = A or C

^‡^For ambiguous sites, amino acid change given for the non-reference base.
